# LncRNA Nron regulates osteoclastogenesis during orthodontic bone resorption

**DOI:** 10.1038/s41368-020-0077-7

**Published:** 2020-05-09

**Authors:** Ruilin Zhang, Junhui Li, Gongchen Li, Fujun Jin, Zuolin Wang, Rui Yue, Yibin Wang, Xiaogang Wang, Yao Sun

**Affiliations:** 1 Department of Implantology, School & Hospital of Stomatology, Tongji University, Shanghai Engineering Research Center of Tooth Restoration and Regeneration, Shanghai, China; 2 School of Biological Science and Medical Engineering, Beihang University, Beijing, China; 3 School of Life Sciences and Technology, Tongji University, Shanghai, China

**Keywords:** Bone, Bone quality and biomechanics

## Abstract

Activation of osteoclasts during orthodontic tooth treatment is a prerequisite for alveolar bone resorption and tooth movement. However, the key regulatory molecules involved in osteoclastogenesis during this process remain unclear. Long noncoding RNAs (lncRNAs) are a newly identified class of functional RNAs that regulate cellular processes, such as gene expression and translation regulation. Recently, lncRNAs have been reported to be involved in osteogenesis and bone formation. However, as the most abundant noncoding RNAs in vivo, the potential regulatory role of lncRNAs in osteoclast formation and bone resorption urgently needs to be clarified. We recently found that the lncRNA Nron (long noncoding RNA repressor of the nuclear factor of activated T cells) is highly expressed in osteoclast precursors. Nron is downregulated during osteoclastogenesis and bone ageing. To further determine whether Nron regulates osteoclast activity during orthodontic treatment, osteoclastic Nron transgenic (Nron cTG) and osteoclastic knockout (Nron CKO) mouse models were generated. When Nron was overexpressed, the orthodontic tooth movement rate was reduced. In addition, the number of osteoclasts decreased, and the activity of osteoclasts was inhibited. Mechanistically, Nron controlled the maturation of osteoclasts by regulating NFATc1 nuclear translocation. In contrast, by deleting Nron specifically in osteoclasts, tooth movement speed increased in Nron CKO mic*e*. These results indicate that lncRNAs could be potential targets to regulate osteoclastogenesis and orthodontic tooth movement speed in the clinic in the future.

## Introduction

Orthodontic tooth movement (OTM) is a process of mechanically induced bone remodelling in which bone resorption occurs on the compression side, and new bone is formed on the tension side.^1^ The formation of osteoclasts on the compression side is the key precondition for tooth movement. Therefore, understanding the role of the biomolecules and signalling pathways involved in osteoclastogenesis during orthodontic treatment is crucial for achieving high-efficiency tooth movement.^2–4^ At present, research mainly focuses on inflammatory cytokines that regulate the formation and activity of osteoclasts.^5,6^ For example, the inflammatory factors IL-12 and IFN-γ significantly inhibit osteoclast formation, while IL-1 and IFN-α promote osteoclast activation.^7^ Noncoding RNAs (ncRNAs), which are functional RNA molecules that are transcribed from DNA but are not translated into proteins, also play crucial roles during osteoclast formation. MiRNA-148a promotes the differentiation of osteoclasts, while miRNA-124 and miRNA-29b exert inhibitory effects on osteoclast formation.^8–10^ Major osteoclastogenic signals are macrophage colony-stimulating factor (MCSF) and receptor activator of nuclear factor-kappa B ligand (RANKL).^4^

As a class of noncoding RNAs that occupy 98.5% of the human genome, the functions of long noncoding RNAs (lncRNAs) have recently attracted increasing attention.^11^ LncRNAs, which contain 200 or more nucleotides, bind to proteins, DNA or RNAs to form functional complexes and play key roles in the regulation of gene expression, translation, transportation, organ development and the occurrence of severe diseases.^11,12^ Recent studies from several teams, including our group, have revealed that lncRNAs participate in osteoblast differentiation, bone development and skeletal diseases.^13–15^ However, limited information is available on the role of lncRNAs during osteoclastogenesis in vivo. Thus, osteoclast formation-related lncRNAs need to be further investigated to gain a better understanding of orthodontic bone resorption.

The long-noncoding RNA repressor of the nuclear factor of activated T cells (NRON) is relatively conserved, and its homologous gene Nron is present in mice.^16^ We found that NRON or Nron expression was obviously decreased in the alveolar bone of elderly and aged mice. In addition, we found that during the differentiation of osteoclasts, Nron expression gradually decreased. Compared with its expression in other cell types in bone, Nron is highly expressed in osteoclast precursor cells. Therefore, we further focused our investigation on whether Nron plays a role in OTM by regulating osteoclast formation. To explore the role of Nron in osteoclast formation in vivo, osteoclastic Nron transgenic (Nron cTG) and knockout (Nron CKO) mice were generated. In addition, Nron interfered with NFAT protein entry into the nucleus.^17^ The NFAT family member NFATc1 functions as a transcription factor during osteoclast formation.^18^ Therefore, we also investigated whether Nron effectively inhibited osteoclastogenesis by regulating NFATc1 in osteoclasts.

## Results

### Nron was negatively correlated with loss of bone mass in alveolar bone

**Fig. 1:**
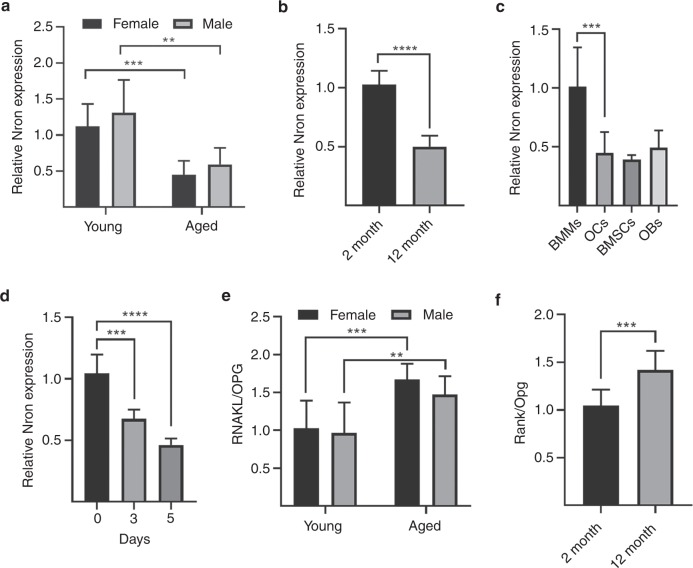
Nron negatively correlates with osteoclast activity in alveolar bone. **a** RT-qPCR analysis of NRON expression in alveolar bone specimens from young and aged individuals; *n* = 8 per group. **b** RT-qPCR analysis of Nron expression in alveolar bone specimens from 2-month-old and 12-month-old WT mice; *n* = 8 per group. **c** RT-qPCR analysis of Nron expression in BMMs, OCs, BMSCs and OBs from 2-month-old WT mice. BMMs, bone marrow-derived macrophages; OCs, osteoclasts; BMSCs, bone marrow stromal cells; OBs, osteoblasts. *n* = 8 per group. **d** RT-qPCR analysis of Nron expression in BMMs from 2-month-old WT mice during osteoclast differentiation; *n* = 6 per group. **e** RT-qPCR analysis of RANKL/OPG expression in alveolar bone specimens from young and aged individuals; *n* = 8 per group. **f** RT-qPCR analysis of Rankl/Opg expression in alveolar bone specimens from 2-month-old and 12-month-old WT mice; *n* = 6 per group. RT-qPCR quantitative reverse transcription polymerase chain reaction. GAPDH and Gapdh were used as endogenous controls in RT-qPCR analysis of human and mouse specimens, respectively. Data are presented as the mean ± SD. *P < 0.05, **P < 0.01, ***P < 0.001, ****P < 0.000 1

First, we detected Nron expression in human alveolar bone specimens collected from young (20–45 years old) and aged individuals (>50 years old) during wisdom tooth extraction. Interestingly, the quantitative reverse transcription polymerase chain reaction (RT-qPCR) results showed that NRON expression in alveolar bone significantly decreased with age (Fig. 1a). We also assessed Nron expression in alveolar bone from 2-month-old and 12-month-old mice. Nron expression was also significantly reduced in aged mice (Fig. 1b). We further examined Nron expression in different cell types in alveolar bone, including bone marrow-derived macrophages (BMMs), osteoclasts (OCs), bone marrow stromal cells (BMSCs) and osteoblasts (OBs). Interestingly, a significant decrease in Nron expression in osteoclasts was noted compared with that of BMM precursor cells (Fig. 1c). Then, we detected Nron expression during osteoclast formation and found that Nron expression decreased significantly (Fig. 1d). Moreover, in alveolar bone collected from humans and mice, the ratio of RANKL/OPG was significantly increased in the aged group (Fig. 1e, f). With ageing, an imbalance in bone remodelling results in osteoporosis. Taken together, these data indicated a negative correlation between Nron and bone mass loss in alveolar bone.

### Nron expression decreased during orthodontic tooth movement

**Fig. 2:**
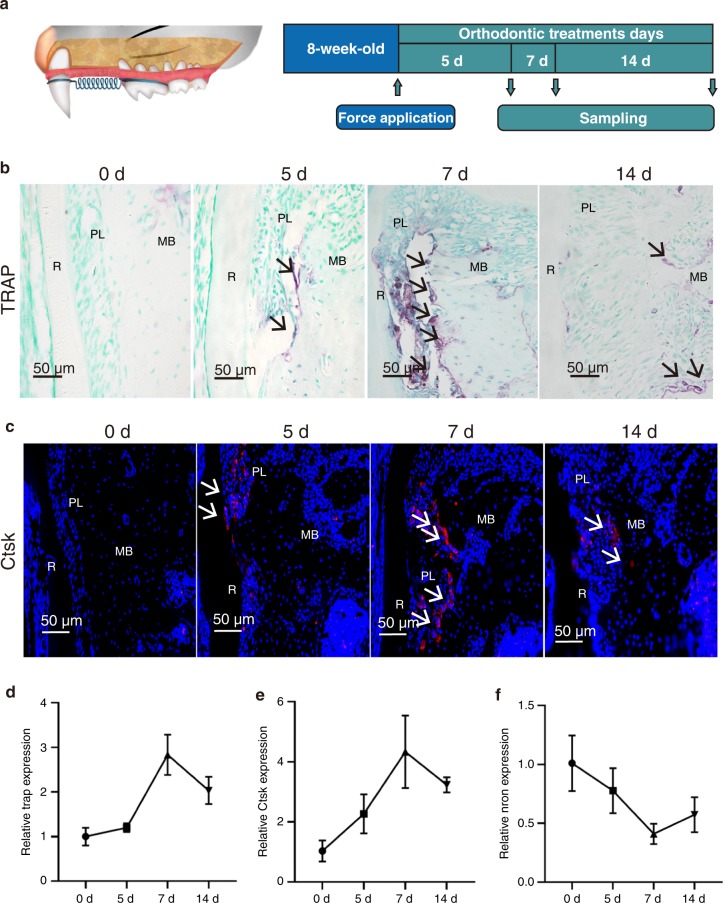
Nron expression decreased during orthodontic tooth movement. **a** Schematic diagram of the OTM model in mice. **b** Representative TRAP staining images of alveolar bone from 2-month-old WT mice under orthodontic tooth treatment. R, root; PL, periodontal ligament; MB, marginal bone. Black arrows indicate osteoclasts. **c** Immunofluorescence staining of CTSK (red)-positive osteoclasts in alveolar bone from 2-month-old WT mice undergoing orthodontic tooth treatment. White arrows indicate CTSK-positive osteoclasts. **d** RT-qPCR analysis of Trap expression in alveolar bone from 2-month-old mice after orthodontic tooth treatment. **e** RT-qPCR analysis of Ctsk expression in alveolar bone from 2-month-old mice after orthodontic tooth treatment. **f** RT-qPCR analysis of Nron expression in alveolar bone from 2-month-old WT mice after orthodontic tooth treatment

Next, an orthodontic model with WT mice was established to verify the relationship between Nron and osteoclast activity in vivo (Fig. 2a). Nron cTG mice displayed higher bone mass than WT mice. The bone volume per tissue volume (BV/TV) and trabecular number (Tb. N) were significantly increased in the femurs of Nron CKO mice (Fig. S3a). During the 14-day orthodontic treatment, TRAP-positive osteoclasts in the compression side of the alveolar bone around the maxillary first molar increased and reached a maximum level on the 7th day (Fig. 2a). CTSK-positive osteoclasts increased during the first 7 days of orthodontic treatment and then decreased, which was consistent with the TRAP staining results (Fig. 2c). The RT-qPCR results showed that TRAP and Ctsk expression in the alveolar bone around the maxillary first molar significantly increased after 7 days of treatment and was subsequently reduced (Fig. 2d, e). In contrast, Nron expression markedly decreased during the first 7 days of orthodontic treatment and then increased (Fig. 2f). In summary, Nron was negatively correlated with osteoclast formation in alveolar bone during orthodontic treatment.

### Nron overexpression inhibited orthodontic tooth movement

**Fig. 3:**
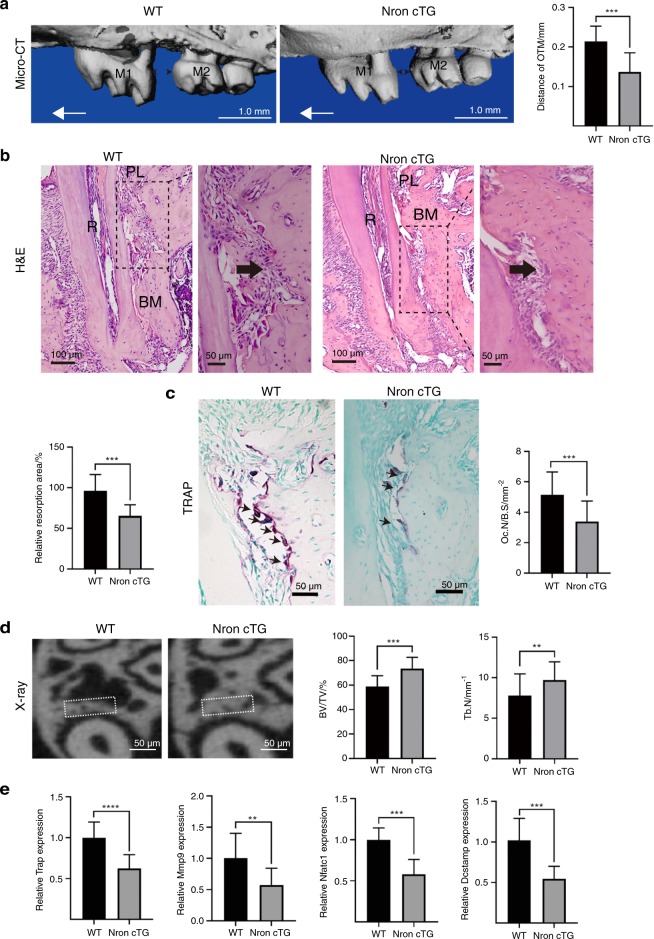
Nron overexpression inhibited orthodontic tooth movement. **a** Three-dimensional reconstruction of the maxilla from 2-month-old WT and Nron cTG mice after 14 days of orthodontic tooth treatment and quantification of OTM distance. M1, first molar; M2, second molar; OTM, orthodontic tooth movement. The red one-way arrow indicates the direction of force; the red two-way arrow indicates the distance of OTM. **b** Representative H&E staining images of alveolar bone from 2-month-old WT and Nron cTG mice after 14 days of orthodontic tooth treatment and quantification of bone resorption. R, root; PL, periodontal ligament; MB, marginal bone. **c** Representative TRAP staining images of alveolar bone from 2-month-old WT and Nron cTG mice after 14 days of orthodontic tooth treatment and quantification of Oc.N/B.S. Oc.S/B.S., osteoclast surface per bone surface. **d** Representative X-ray images of alveolar bone of 2-month-old WT and Nron cTG mice after 14 days of orthodontic tooth treatment and quantification of BV/TV and Tb.N. BV/TV, bone volume per total volume;Tb.N., trabecular bone number. **e** RT-qPCR analysis of *Trap, Mmp9, Nfact1* and *Dcstamp* expression in alveolar bone from 2-month-old WT and Nron cTG mice after orthodontic tooth treatment. *P < 0.05, **P < 0.01, ***P < 0.001, ****P < 0.000 1. Data are presented as the mean ± SD; *n* = 6 per group

To further examine the role of Nron in orthodontic bone resorption, we constructed osteoclastic Nron transgenic (Nron cTG) mice, and Nron overexpression was confirmed by RT-qPCR in osteoclasts isolated from these mice (Fig. S1). After application of orthodontic force to the teeth for 14 days, the microcomputed tomography (micro-CT) results showed significantly decreased movement of the left maxillary first molar in Nron cTG mice compared with their WT littermates (Fig. 3a). We also found that bone resorption was alleviated and that the resorption area on the compression side of the left maxillary first molar distal root was obviously reduced in Nron cTG mice, as observed by H&E staining (Fig. 3b). In addition, the number of TRAP-positive osteoclasts was reduced in Nron cTG mice, and the bone resorption parameter Oc. S/BS was significantly decreased (Fig. 3c). Then, we measured the bone mass of the compression side of the root bifurcation and found that it was notably increased, and the BV/TV and Tb. N of this area were significantly increased in Nron cTG mice (Fig. 3d). We further examined the expression of bone resorption-related markers, including *Trap, Mmp9, Nfatc1* and *Dcstamp*, in the alveolar bone in response to orthodontic force and found a significant reduction in all these markers when Nron was overexpressed (Fig. 3e).

### Nron inhibited osteoclast differentiation by reducing nuclear import of NFATc1

**Fig. 4:**
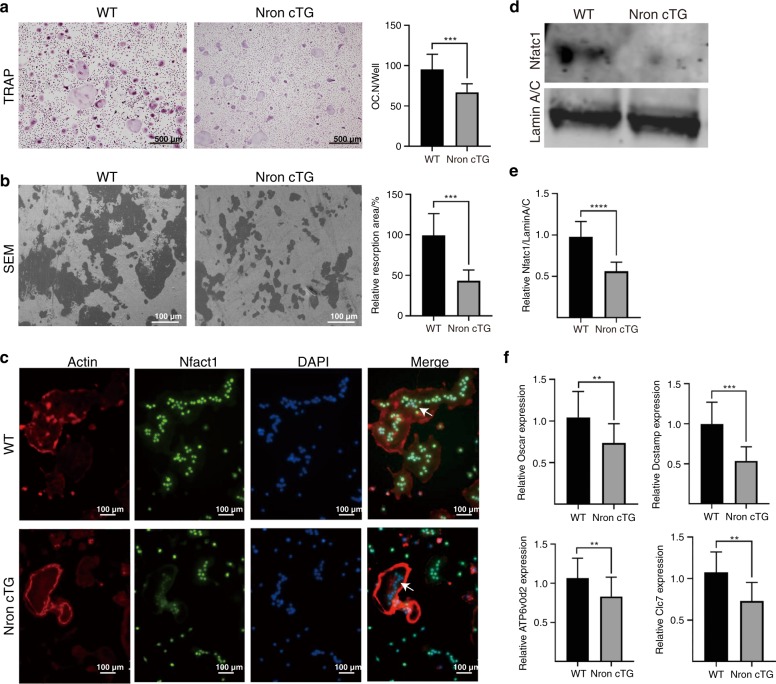
Nron inhibits osteoclast differentiation by reducing the nuclear import of Nfatc1. **a** Representative TRAP staining images of WT and Nron cTG BMMs induced with osteoclastic differentiation medium for 6 days and quantification of Trap-positive osteoclasts. BMMs, bonemarrow-derived macrophages. **b** SEM images of the bone resorption plate and quantification of the resorption area. SEM scanning electronic microscope. **c** Immunofluorescence staining of actin and Nfatc1 in WT and Nron cTG osteoclasts. Green signals indicate Nfatc1 located in the nucleus. **d** Expression of Nfatc1 in osteoclast nuclei from WT and Nron cTG mice was analysed by western blot. **e** Quantification of the Nfatc1 protein levels in **d**. **f** RT-qPCR analysis of *Oscar, Dcstamp, Atp6vod2* and *Clc7* expression in osteoclasts from the two groups. *P < 0.05, **P < 0.01, ***P < 0.001, ****P < 0.000 1. Data are presented as the mean ± SD; *n* = 6 per group

To further explore the mechanism of Nron in osteoclastogenesis, BMMs isolated from alveolar bone were induced with M-CSF and RANKL for 6 days. In the Nron cTG group, fewer BMMs differentiated into osteoclasts, and the number of TRAP-positive osteoclasts significantly decreased compared with that of WT mice (Fig. 4a). To examine osteoclast resorption activity, bone resorption experiments were implemented. A reduced bone resorption area was observed when Nron was overexpressed in osteoclasts (Fig. 4b). For a deeper understanding of Nron regulation in osteoclasts, we performed immunofluorescence staining and found that Nfatc1 nuclear localisation was markedly reduced in Nron cTG osteoclasts (Fig. 4c). Nfatc1 protein levels in isolated osteoclast nuclei were assessed by western blotting, and Nfatc1 protein levels were obviously reduced in Nron cTG osteoclasts compared with WT osteoclasts (Fig. 4d, e). Additionally, reduced expression of Nfatc1-related molecules, including *Oscar, DCSTAMP*, *ATP6vod4* and *CIC7*, was detected in Nron cTG osteoclasts by RT-qPCR (Fig. 4f). In summary, Nron inhibited the nuclear import of Nfatc1 and subsequently inhibited osteoclast maturation.

### Accelerated orthodontic tooth movement rate in osteoclastic Nron knockout mice

**Fig. 5:**
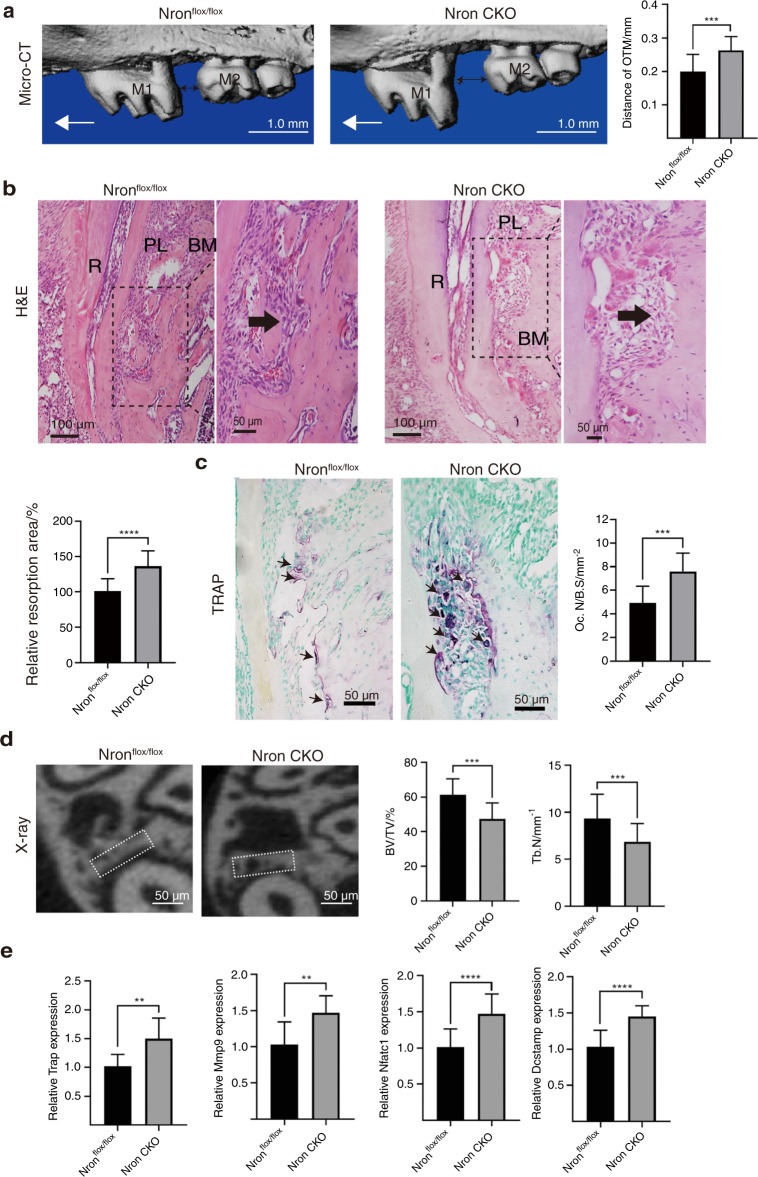
Accelerated orthodontic tooth movement rate in osteoclastic Nron knockout mice. **a** Three-dimensional reconstruction of the maxilla from 2-month-old Nron^flox/flox^ and Nron CKO mice after 14 days of orthodontic tooth treatment and quantification of OTM distance. M1, first molar; M2, second molar; OTM, orthodontic tooth movement. The red one-way arrow indicates the direction of force; the red two-way arrow indicates the distance of OTM. **b** Representative H&E staining images of alveolar bone from 2-month-old Nron^flox/flox^ and Nron CKO mice after 14 days of orthodontic tooth treatment and quantification of bone resorption. R, root; PL, periodontal ligament; MB, marginal bone. **c** Representative TRAP staining images of alveolar bone from 2-month-old Nron^flox/flox^ and Nron CKO mice after 14 days of orthodontic tooth treatment and quantification of Oc.N/B.S. Oc.S/B.S., osteoclast surface per bone surface. **d** Representative X-ray images of alveolar bone of 2-month-old Nron^flox/flox^ and Nron CKO mice after 14 days of orthodontic tooth treatment and quantification of BV/TV and Tb.N. BV/TV, bone volume per total volume; Tb.N., trabecular bone number. **e** RT-qPCR analysis of *Trap, Mmp9, Nfatc1* and *Dcstamp* expression in alveolar bone from 2-month-old Nron^flox/flox^ and Nron CKO mice after orthodontic tooth treatment. *P < 0.05, **P < 0.01, ***P < 0.001, ****P < 0.000 1. Data are presented as the mean ± SD; *n* = 6 per group

To further verify the regulatory role of Nron in orthodontic bone remodelling, osteoclastic Nron knockout (Nron CKO) mice were constructed. Nron expression decreased dramatically in osteoclasts isolated from osteoclastic Nron CKO mice (Fig. S2). Nron CKO mice displayed lower bone mass than Nron^flox/flox^ mice. The BV/TV and Tb. N were significantly decreased in the femurs of Nron CKO mice (Fig. S3b). After 14 days of orthodontic force, the OTM distance of Nron CKO mice increased significantly (Fig. 5a). Large alveolar bone absorption areas on the compression side of the left maxillary first molar distal root were observed in Nron CKO mice (Fig. 5b). In addition, enhanced alveolar bone resorption activity in Nron CKO mice was confirmed by TRAP staining, and the osteoclastic bone resorption parameter Oc. S/BS was also significantly increased (Fig. 5c). The X-ray results showed that Nron CKO mice had less bone mass on the compression side of the root furcation than Nron^flox/flox^ mice. Then, we measured the BV/TV and Tb. N of this region and found significant decreases (Fig. 5d). Significantly increased expression of *Trap*, *Mmp9*, *Nfatc1* and *Dcstamp* was detected in alveolar bone in response to orthodontic treatment when Nron was knocked out in osteoclasts (Fig. 5e). Osteoclasts of Nron CKO mice showed increased numbers of nuclei and increased NFATc1 (Fig. S5). In summary, Nron knockout in osteoclasts accelerated the orthodontic tooth movement rate.

## Discussion

During bone resorption in OTM, the regulation of osteoclast differentiation is a complex process involving multiple cytokines (TGF-β, M-CSF, IL-1, IL-6, IL-11 and TNF-α) and the RANKL-RANK pathway.^19^ In addition to protein-coding genes, noncoding RNAs are also involved in osteoclast formation. Currently, investigations of ncRNAs involved in osteoclastogenesis are mainly limited to miRNAs.^20^ However, the vast majority of noncoding RNAs are lncRNAs that act as signal and scaffold molecules to regulate cell fate.^21^ The abnormal expression or dysfunction of lncRNAs is closely related to human diseases, such as cancer and degenerative diseases.^22,23^ In addition, lncRNAs have stronger tissue specificity and temporal-spatial expression specificity than miRNAs. To date, studies of lncRNAs in the skeletal system are generally rare and mainly focus on bone formation and bone disorders, such as osteoporosis and osteoarthritis.^24–27^ Preliminary studies showed that thousands of lncRNAs are differentially expressed in a stage-specific manner during osteoclastogenesis.^28^ For example, the lncRNA DANCR participates in osteoclast differentiation by inducing interleukin-6 (IL-6) and tumour necrosis factor-alpha (TNF-α) expression.^29^ The lncRNA MALAT1 regulates the RANK/RANKL/OPG pathway to control osteoclast activity in vitro.^30^ However, there is limited knowledge of the regulatory functions of lncRNAs during osteoclast differentiation in vivo.

Nron functions in HIV-1 disease, heart failure and tumour cell proliferation.^16,31,32^ Nron is expressed in peripheral blood mononuclear cells, which are the major source of osteoclasts.^33^ Therefore, this study focused on the role of Nron in the differentiation of osteoclasts using an orthodontic bone remodelling model. Among bone cell types, osteoclast precursors specifically expressed high levels of Nron. In addition, Nron expression gradually decreases during osteoclast differentiation. As the alveolar bone ages, Nron expression is reduced, which is accompanied by an increase in the loss of bone mass. Therefore, we further explored the effect of Nron on osteoclast differentiation by constructing conditional overexpression and knockout mouse models. When Nron was overexpressed in osteoclasts, osteoclast activity was blocked, and the orthodontic tooth movement rate decreased accordingly. In contrast, when Nron was knocked out in osteoclasts, osteoclast formation increased, and alveolar bone resorption and orthodontic tooth movement rates increased. These findings indicate that Nron expression is negatively correlated with osteoclast formation. Thus, lncRNAs such as Nron could be considered new targets for regulating osteoclast activity.

Nuclear factor of activated T cells (NFAT) is a transcription factor that was first identified in nuclear extracts from activated T cells. The NFAT family plays an important role in cancer invasion, migration, and angiogenesis.^34^ Nron binds to NFAT kinase and IQGAP1 to form an RNA-protein complex that ultimately affects the transport of NFAT into the nucleus.^17^ NRON suppresses vein endothelial cell proliferation and invasion through the inhibition of NFAT activity.^35^ Knockdown of NRON led to a substantial increase in NFAT dephosphorylation and nuclear translocation.^36^ In addition, as a transcription factor, the NFAT family member NFATc1 plays a crucial role in the formation of osteoclasts.^18^ RANKL-activated NFATc1 regulates osteoclast formation by promoting the transcription of TRAP, CTSK, the calcitonin receptor and the osteoclast-associated receptor (OSCAR).^18,37–39^ Therefore, we further tested whether Nron inhibited the nuclear translocation of NFATc1 in osteoclasts. Both immunofluorescence staining and western blot results confirmed that Nron overexpression inhibited NFATc1 nuclear entry in osteoclasts in vitro. This finding indicates that Nron inhibits osteoclastogenesis by regulating the import of NFATc1 into osteoclastic nuclei, which affects the differentiation of osteoclasts.

This study is the first to provide evidence that a functional lncRNA inhibits osteoclastogenesis during alveolar bone remodelling. Downregulation of Nron in osteoclasts promotes osteoclast formation and OTM. This investigation demonstrates the likely utility of lncRNAs as new therapeutic targets to regulate tooth movement during orthodontic treatment.

## Materials and methods

### Generation of osteoclastic Nron transgenic mice

Osteoclastic Nron transgenic (Nron cTG) mice on a C57BL/6J background were generated by Cyagen Biosciences (Guangzhou, China), and a schematic diagram of the transgenic mouse construction is shown in Fig. S1. In brief, the mouse Ctsk promoter and Nron cDNA were subcloned into the pPB[Exp]-CAG plasmid to construct the pPB[Exp]-CAG > Ctsk-Nron vector. After the osteoclast-specific expression of the vector was verified in vitro, the linearised pPB[Exp]-CAG > Ctsk-Nron plasmid was microinjected into C57BL/6J oocytes. Then, the oocytes were transferred into pseudopregnant C57BL/6J mice. Finally, two pups out of 80 were identified as Nron-cTG mice. The line with >10-fold overexpression of Nron was used for further studies. The genotyping PCR primer sequences are listed in Table S1. Wild-type (WT) C57BL/6J mice were used as controls.

### Generation of osteoclastic Nron knockout mice

Nron-floxed mice were generated on a C57BL/6J background by the Shanghai Research Center for Model Organisms (Shanghai, China) (Fig. S2). Briefly, after constructing a targeting vector containing a 7.6 kb 5′-homologous arm, 4.3-kb flox region, PGK-Neo-polyA, 4.0-kb 3′-homologous arm and MC1-TK-polyA negative screening marker, the linearised vector was electrotransfected into embryonic cells. Nested long-range PCR was used to screen for three positive clones. Then, the positive clones were injected into blastocysts of C57BL/6J mice, and four positive F0 chimeric mice were obtained. After removing the PGK-Neo cassette by crossing F0 chimeric mice with Rosa26-FlpE knock-in mice, floxed F1 mice were generated. Finally, Nron-floxed mice were crossed with Ctsk-Cre mice to obtain osteoclastic Nron knockout (Nron CKO) mice. Cre-dependent Nron knockout mice without Cre were used as littermate controls. The genotyping PCR primer sequences are listed in Table S1.

### Application of orthodontic devices

The orthodontic device was applied according to a previously described protocol.^40^ In short, after anaesthetising 8-week-old male mice with 10% pentobarbital (0.05 mL per 10 g), we fixed the mice on the operating table by using tape to immobilise the limbs. Then, we used a mini mouth gap to open the mouth. After cleaning the teeth with 75% alcohol wipes, we attached a 4-mm nickel-titanium coil spring (Smart Technology, China) to the left maxillary first molar and left maxillary incisor using a 0.1-mm diameter steel wire. Self-etch adhesive (3 M, USA) was used to handle the surface of the left maxillary incisor for 30 s and was illuminated with curing lights for another 30 s. Finally, light-cured resin (3 M, USA) was applied on the left maxillary incisor to fix the steel wire. The spring applied an approximate force of 35 g to the tooth as measured by a dynamometer (Chatillon, USA). The contralateral side of the maxilla served as the control. After model establishment, the mice were fed a soft diet. Then, 5, 7 and 14 days after application of the orthodontic device, the mice were killed, and alveolar bone tissues were harvested.

### Microcomputed tomography analysis

After heart perfusion with 20 mL phosphate-buffered solution (PBS, BBI, China) and 20 mL paraformaldehyde (PFA, Aladdin, China), the left maxillae were isolated from the mice and fixed in 4% PFA (Aladdin, China) for 2 days. Then, the left maxillae that had been collected from mice were scanned by μCT-50 (Scanco Medical, Switzerland) with a 10-μm scan resolution (10 μm per slice). A three-dimensional representative reconstruction image of the left maxillary bone was obtained with the horizontal morphology of the buccal bone plate ridge as a reference. The distance between the midpoint of the distal edge ridge of the left maxillary first molar and the midpoint of the proximal edge ridge of the left maxillary second molar was measured and regarded as the OTM distance. The bone mass analysis of the alveolar bone around the maxillary first molar was limited to the region of the compression side under the root furcation near the distal root of the maxillary first molar. Fifteen slices were reconstructed for statistical analysis. Representative X-ray images were taken at the 15th scan layer under the root furcation. The threshold value of the micro-CT was set from 212 to 1 000.

### Haematoxylin and eosin staining

The left maxilla was harvested, fixed in 4% PFA (Aladdin, China) and then decalcified in 10% ethylenediaminetetraacetic acid (EDTA, BBI, China) at room temperature for 28 days. The decalcification solution was replaced every 2 days. After dehydration in an automatic dehydrator (Thermo, USA), the specimens were embedded in paraffin and sectioned at a thickness of 4 μm using a Microm HM325 (Thermo, USA). Then, an H&E staining kit (MXB, China) was used for staining according to the manufacturer’s protocol.

### TRAP staining

For TRAP staining, the cells were rinsed with PBS twice and fixed with 4% PFA (Aladdin, China) for 30 min at room temperature. Then, the cells were incubated with a tartrate-resistant acid phosphatase (TRAP) staining kit (Sigma, USA) according to the manufacturer’s instructions. Briefly, the cells were incubated in TRAP solution at 37 °C for 15 min.

After dewaxing in xylene and gradient alcohol, the sections were incubated in TRAP solution at 37 °C for 15 min, counterstained with 1% methyl green (Sigma, USA) for 10 min at room temperature and then rinsed with ethyl alcohol. Finally, the sections were imaged using a Nikon microscope (Nikon, Japan), and osteoclast quantification was performed using ImageJ software (version 1.8.0).

### Immunofluorescence assay

After decalcification, the mouse maxillae were embedded in optimal cutting temperature compound (OCT; Tissue-Tek, USA) and sliced at a thickness of 8 μm using a CM1850 (Leica, USA). Then, the sections were incubated with hyaluronidase (HA; Sigma, USA) for 1 h at 37 °C, followed by incubation with goat serum (MXB, China) for 1 h. After incubation with anti-CTSK antibody (ab19027; dilution 1:400; Abcam, USA) overnight at 4 °C, the sections were incubated with Alexa Flour 586 IgG (ab 150088; dilution 1:1 000; Abcam, USA) for 1 h. Finally, the sections were counterstained with DAPI (Sigma, USA) for 10 min. For immunofluorescent cellular assays, before staining, the cells were fixed with 4% PFA (Aladdin, China) for 30 min and incubated with 0.1% Triton X-100 (BBI, China) for 20 min. Then, goat serum (MXB, China) was used to block the cells. Subsequently, the cells were co-incubated with anti-Nfatc1 antibody (ab2722; dilution 1:400; Abcam, USA) and anti-actin antibody (ab8227; dilution 1:500, Abcam, USA) overnight at 4 °C. Alexa Flour 488 IgG (ab150113; dilution 1:1 000; Abcam, USA) and Alexa Flour 586 IgG (ab 150088; dilution 1:1 000; Abcam, USA) were used to visualise Nfatc1 and actin in the cells. Finally, the nuclei were counterstained with DAPI (Sigma, USA). An inverted fluorescence microscope (Nikon, Japan) was used to obtain images.

### Western blot assay

After rinsing with ice-cold PBS, the cells were scraped from plates using SDS lysis buffer (Beyotime, China) containing protease inhibitors (Beyotime, China). Then, the cells were lysed and centrifuged at 12 000 × *g* for 10 min at 4 °C to collect the supernatant. Protein concentrations were measured by using a BCA protein assay kit (Beyotime, China). Proteins were separated by SDS-PAGE and transferred to PVDF membranes (Millipore, USA). The membranes were blocked and then incubated with anti-Nfatc1 antibody (ab2722; dilution 1:400; Abcam, USA) and anti-lamin A/C antibody (ab108922; dilution 1:400, Abcam, USA). After incubation with the secondary antibodies for 1 h, a chemiluminescence reagent (Millipore, USA) was used to visualise the blots. Quantity One software (Bio-Rad, USA) was utilised to quantify the band densities.

### Quantitative reverse transcription polymerase chain reaction

Total RNA was isolated from alveolar bone tissue or cells using TRIzol reagent (Invitrogen, USA). After 30 min at 4 °C, chloroform was added to the TRIzol solution. Then, centrifugation was performed at 12 000 × *g* and 4 °C for 20 min, and the supernatant was obtained. After mixing with the same volume of isopropanol, the supernatant was centrifuged at 10 000 × *g* for 15 min at 4 °C to obtain the RNA pellet. In addition, 75% ethyl alcohol diluted with DEPC-treated water was used to wash the RNA pellet twice, with centrifugation at 8 000 × *g* for 10 min at 4 °C. After dissolving the RNA pellet in 20 μL DEPC-treated water, the RNA concentration was measured by a spectrophotometer (GE, USA), and 1 000 ng of RNA was reverse transcribed into cDNA in a 20 µL reaction volume using the Transcriptor First Strand cDNA Synthesis Kit (Roche, Germany) according to the manufacturer’s instructions. Then, RT-qPCR was conducted with a SYBR Premix Ex Taq II kit (Takara, China) in a 10-μL volume. The primer sequences used in this study are listed in Table S2. All primers were purchased from Sangon Biotech (China). The 2^−ΔΔCT^ method was used for data analyses.

### Isolation of BMSCs and OBs from alveolar bone

After 8-week-old mice were killed, the alveolar bone cleared of connective tissue was isolated. The bone was cut into pieces after repeated washing with PBS buffer and digested within 0.1% type I collagenase at 37 °C for 180 min. The filtrate was centrifuged at 1 200 r·min^−1^ for 6 min. The supernatant was discarded. The entire culture was suspended, precipitated and inoculated in a 10-cm dish. The culture dish was placed in a 37 °C, 5% CO_2_ incubator, and the cells were allowed to adhere to obtain primary BMSCs. The cell concentration was adjusted to 1 × 10^6^ cells per mL, and the cells were inoculated into a six-well plate. After the cells adhered to the dish and grew to 80%–90% confluence, the osteogenic induction solution for OBs was added.

### Isolation of BMMs and OCs from the mandible

The mandibular bone marrow tissue was added to a α-MEM cell culture solution with a syringe, centrifuged and resuspended in α-MEM cell culture solution containing 10% foetal bovine serum, and inoculated in a 10-cm dish. The tissue was incubated at 5% CO_2_ for 24 h at 37 °C. M-CSF (50 ng·mL^−1^) was cultured for 72 h to remove non-adherent cells. Cell culture fluid containing 50 ng·mL^−1^ M-CSF and 100 ng·mL^−1^ RANKL was used to induce osteoclast differentiation. After 48 h, the cell culture medium was changed.

### Osteoclast differentiation in vitro

Primary bone marrow-derived macrophages (BMMs) were isolated from the femurs of 4-week-old mice. First, the bone marrow cells were cultured in α-MEM (HyClone, USA) containing 10% foetal bovine serum (FBS; Gibco, USA) and 1% penicillin-streptomycin (Sigma, USA) for 48 h. Then, the unattached cells were collected, and red blood cell lysis buffer (BOSTER, China) was used to lyse the erythrocytes. After washing with PBS and centrifuging, the cells were resuspended in α-MEM growth medium (HyClone, USA) containing 50 ng·mL^−1^ M-CSF and then seeded in 6-well cell culture plates at 6 × 10^5^ cells per well or 24-well cell culture plates at 3 × 10^6^ cells per well for 48 h. Subsequently, the cells were cultured in osteoclast differentiation medium (α-MEM growth medium + 10% FBS + 50 ng·mL^−1^ M-CSF + 100 ng·mL^−1^ RANKL), and the medium was renewed every 2 days for a total of 6 days. TRAP-positive multinucleated osteoclasts were identified by using a TRAP staining kit (Sigma, USA). Osteoclast resorption pit assays were performed by culturing cells on 24-well bone resorption plates (Immunodiagnostic Systems, UK) at 6 × 10^5^ cells per well for 8 days in the presence of 50 ng·ml^−1^ M-CSF and 100 ng·mL^−1^ RANKL. Subsequently, the plates were dried in air, and images were obtained using a scanning electron microscope (Hitachi, Japan). All cells were maintained in a 5% CO_2_ humidified incubator at 37 °C.

### Statistical analysis

All statistical analyses were performed with SPSS software, version 16.0. The data are represented as the mean ± SD of independent samples. Significant differences between 2 groups were determined by Student’s *t*-test. A *P*-value < 0.05 was considered statistically significant. Three experiments were repeated independently with similar results for all histomorphological staining.

### Study approval

All animals were bred according to the National Institutes of Health’s Guide for the Care and Use of Laboratory Animals. This study was approved by the Ethics Committee and the Institutional Animal Care and Use Committee of Tongji University (2016-TJ2016005). Human alveolar bone samples from 32 patients (between 20 and 90 years of age) were collected from patients with wisdom tooth extraction via a boning operation at Tongji University Hospital of Stomatology. We obtained written informed consent from all participants prior to inclusion in the study.

## Supplementary information

Supplementary Information

## Data Availability

The data that support the findings of this study are available from the authors upon reasonable request.
