# Supplementary material for: LncRNA Nron regulates osteoclastogenesis during orthodontic bone resorption
Source: Int J Oral Sci. 2020 May 9;12:14. doi: 10.1038/s41368-020-0077-7 (PMC7210890; doi:10.1038/s41368-020-0077-7)

**Supplementary information**

**Title:** LncRNA Nron Regulates Osteoclastogenesis During Orthodontic Bone Resorption

**Authors**: Ruilin Zhang^1^, Junhui Li^1^, Gongchen Li^1^, Fujun Jin^2^, Zuolin Wang^1^, Yibin Wang^3^, Rui Yue^3^, Xiaogang Wang^2*^, and Yao Sun^1*^

**Affiliations：**

1, Department of Implantology, School & Hospital of Stomatology, Tongji University, Shanghai Engineering Research Center of Tooth Restoration and Regeneration, Shanghai, China

2, School of Biological Science and Medical Engineering, Beihang University, Beijing, China

3, School of Life Sciences and Technology, Tongji University, Shanghai, China

***Correspondence should be addressed to:**

Yao Sun, D.D.S, Ph.D.

Department of Implantology, School of Stomatology, Tongji University

Shanghai Engineering Research Center of Tooth Restoration and Regeneration.

399 Middle Yanchang Road, Shanghai, China. Zip code: 200072

Email: [yaosun@tongji.edu.cn](mailto:yaosun@tongji.edu.cn)

or

Xiaogang Wang, M.D., Ph.D.

School of Biological Science and Medical Engineering,

Beihang University, Beijing China. Zipcode: 100191,

E-mail: xiaogangwang@buaa.edu.cn

**
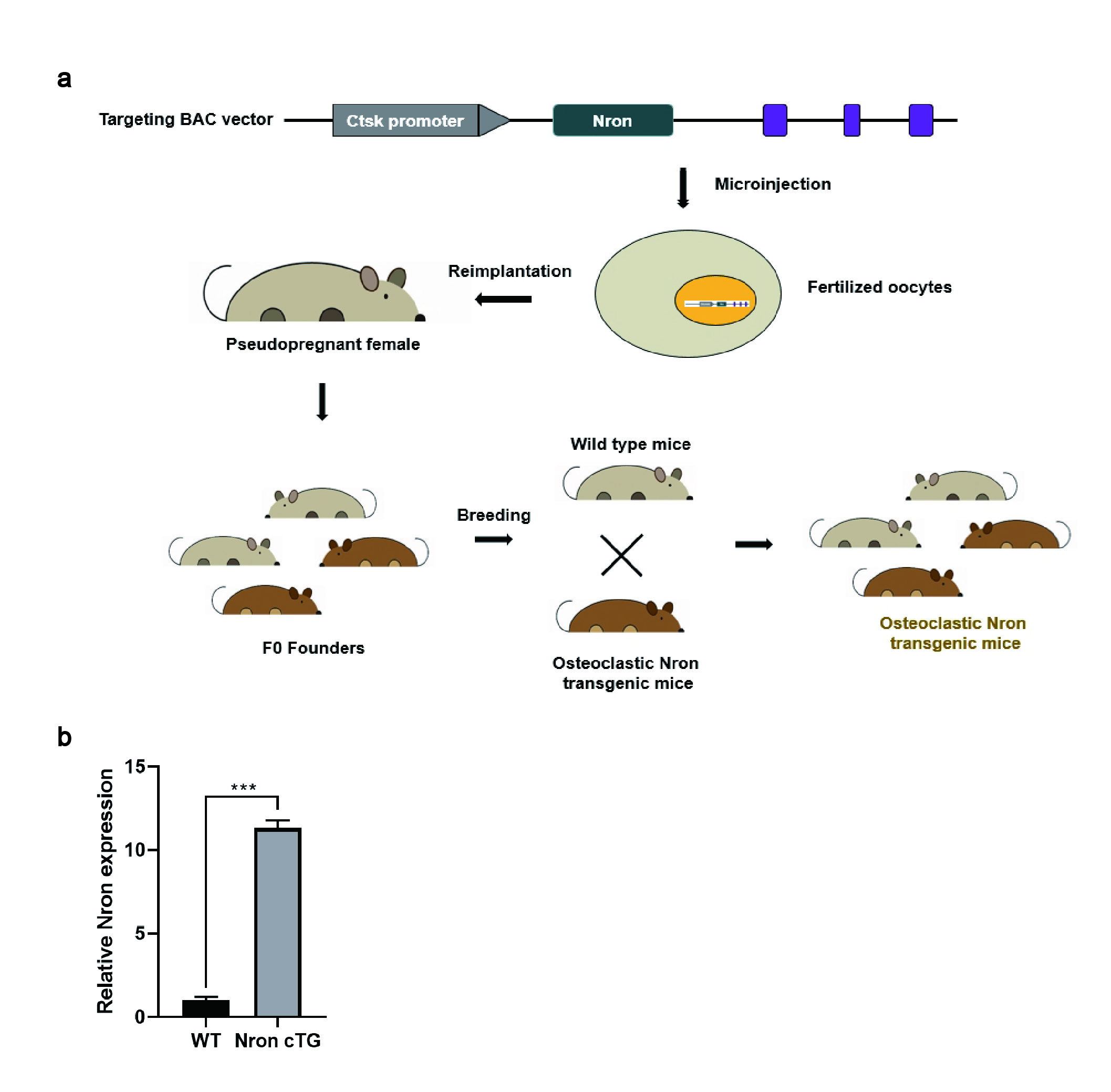
 Fig. S1** Generation of osteoclastic Nron transgenic mice. **a** Schematic illustration of the generation of osteoclast-specific Nron transgenic mice. Briefly, mouse Ctsk promote and Nron cDNA were subcloned into the the pPB[Exp]-CAG plasmid to constructed a pPB[Exp]-CAG>Ctsk-Nron vector. The osteoclast specific expression of the vectors were verified and then the linearized pPB[Exp]-CAG>Ctsk-Nron plasmid was microinjected into C57BL/6J oocytes. Afterward, the oocytes were transferred into pseudopregnant and two from 80 pups were identified as osteoclastic Lnc-Nron transgenic mice. **b** RT-qPCR analysis of Nron expression levels in osteoclasts isolated from 2-month-old WT and Nron cTG mice. n = 4 per group. **P* < 0.05, ***P* < 0.01, ****P* < 0.001, *****P* < 0.0001. Data are presented as the mean ± SD.


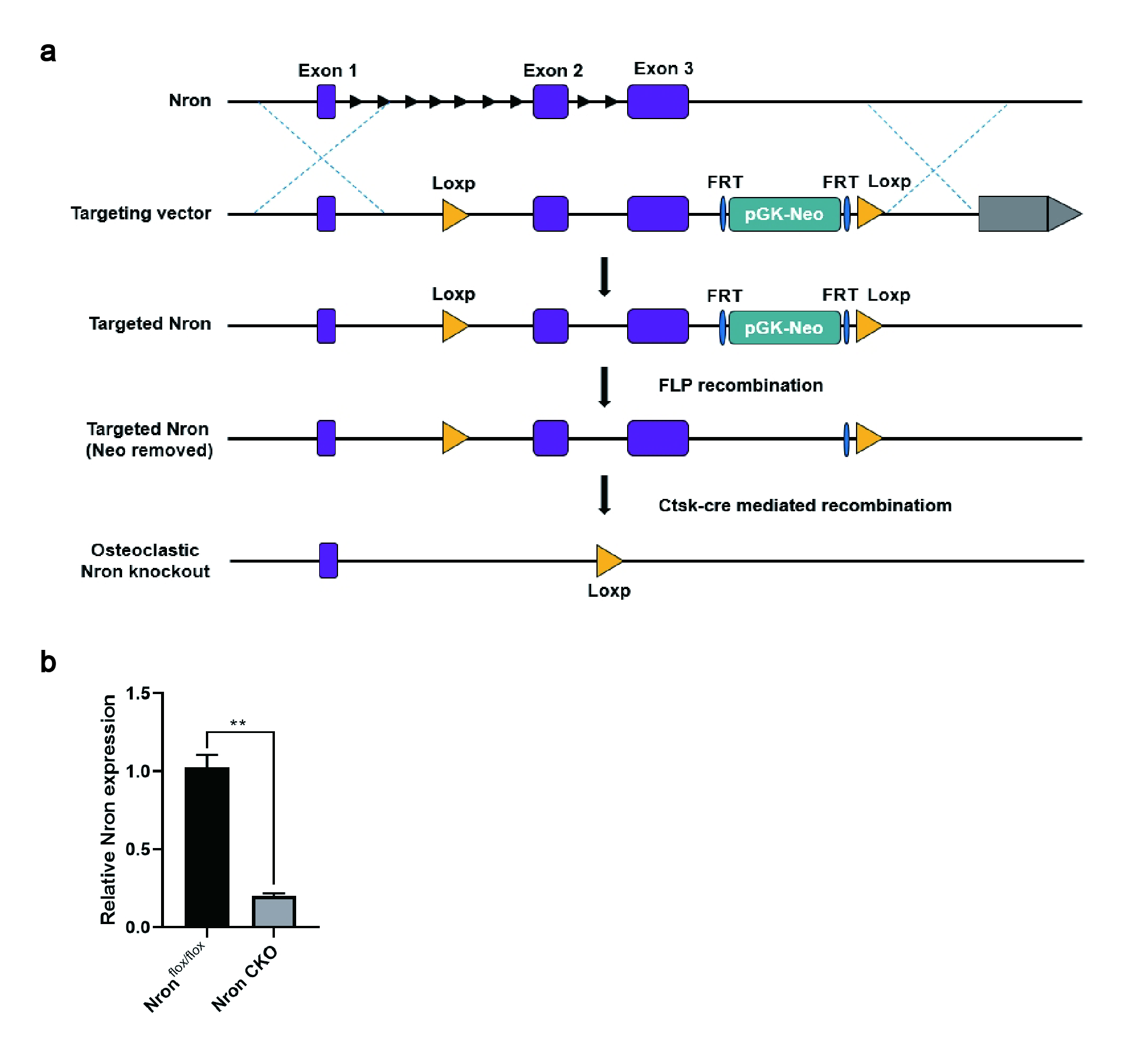


**Fig. S2** Generation of osteoclastic Nron knockout mice. **a** Schematic illustration of the generation of osteoclastic Nron knockout mice. In brief, through infusion method, a targeting vector was constructed and it contains 7.6 kb 5’-homologous arm, 4.3 kb flox region, PGK-Neo-polyA, 4.0 kb 3’-homolohous arm and MC1-TK-polyA negative screening marker. After linearization, the vector was transfected into embryonic cells by electric. With medicine screen of G418 and Ganc, 144 resistant clones were obtained. Then three positive clones were identified using nested long-range PCR. Finally, the positive clones were into blastocysts of C57BL/6J mice and four positive F0 chimeric mice were obtained. To generate the floxed F1 mice, Rosa26-FlpE knock-in mice were crossed with F0 chimeric mice. **b** RT-qPCR analysis of Nron expression levels in osteoclasts isolated from 2-month-old Nron^flox/flox^ and Nron CKO mice. n = 4 per group. **P* < 0.05, ***P* < 0.01, ****P* < 0.001, *****P* < 0.0001. Data are presented as the mean ± SD.


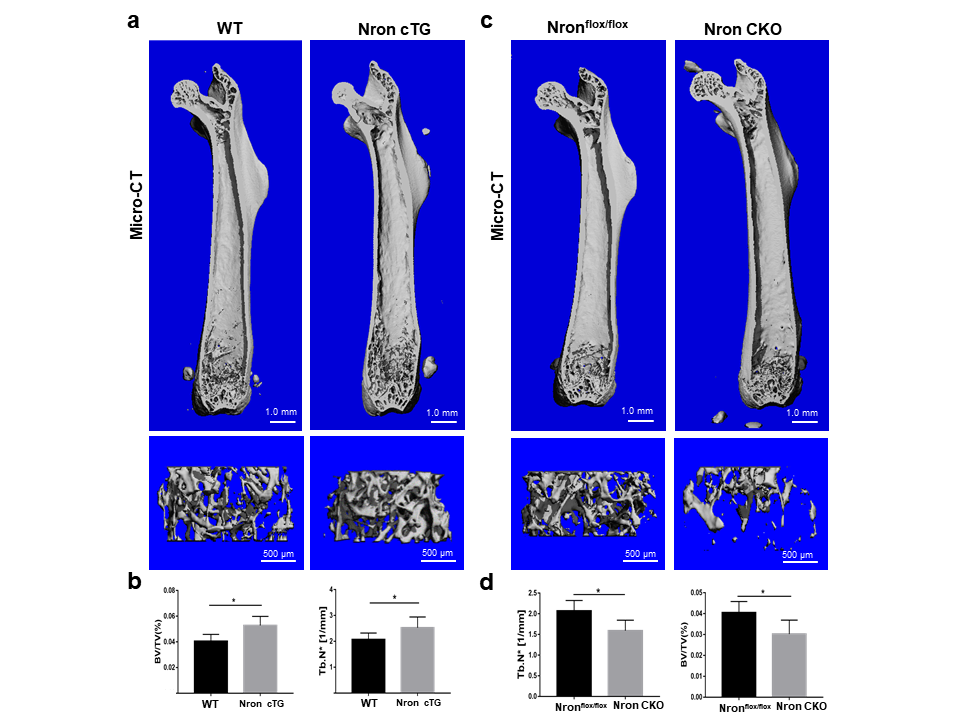


**Fig. S3** Quantitative microtomography (μCT) analysis of the femurs of Nron cTG mice and Nron CKO mice. **a,b** Nron cTG mice displayed higher bone mass as compared with WT mice. The bone volume per tissue volume (BV/TV) and trabecular number (Tb.N) were significantly increased in the femurs of Nron CKO mice. **c,d** Nron CKO mice displayed lower bone mass as compared with Nron^flox/flox^ mice. The bone volume per tissue volume (BV/TV) and trabecular number (Tb.N) were significantly decreased in the femurs of Nron CKO mice


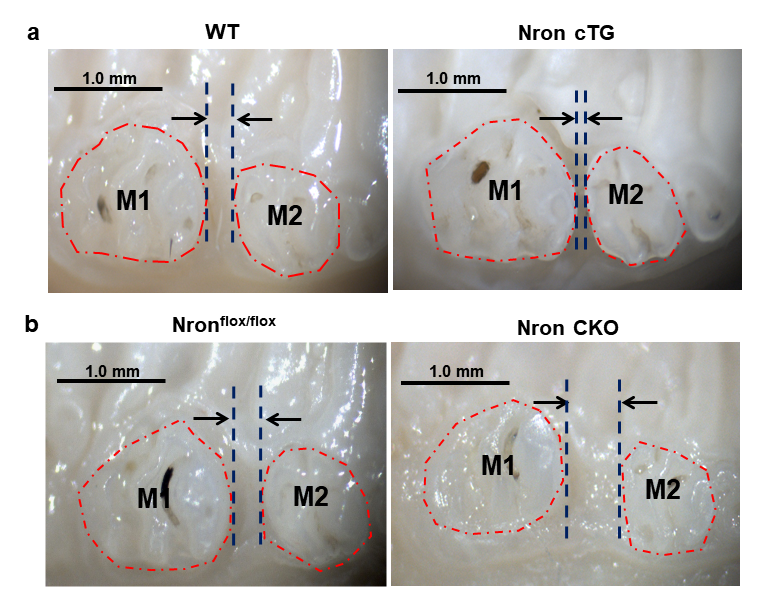


**Fig. S4** The gross pictures of orthodontic samples examined by stereomicroscope had been showed in the supplementary files. The maxillary first molar in Nron cTG mice showed less moving distance than WT littermates while that in Nron CKO mice showed more moving distance.


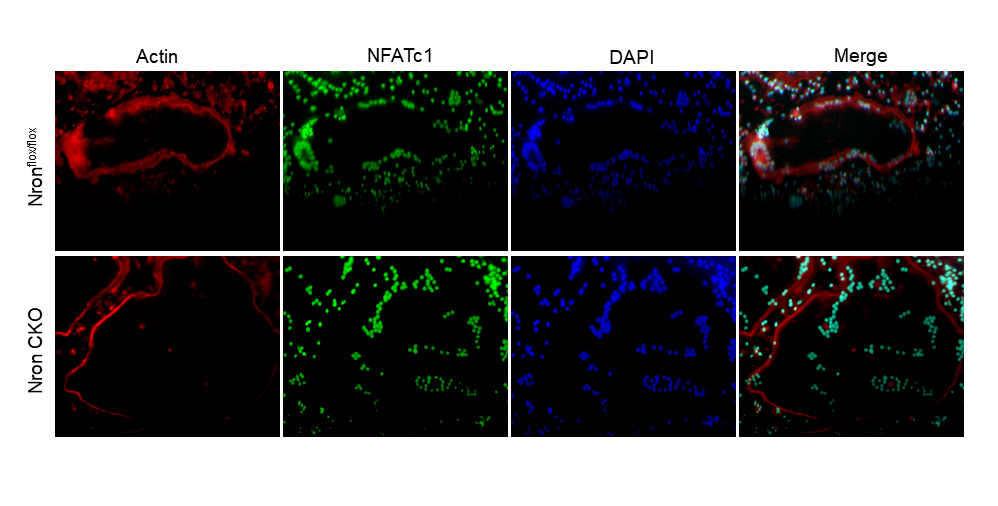


**Fig. S5** Immunofluorescence staining of Actin and Nfatc1 of Nron^flox/flox^ and Nron CKO osteoclasts. Green signals indicate Nfatc1 located in nucleus. Osteoclasts of Nron CKO mice showed more nucleuses and increased NFATc1.


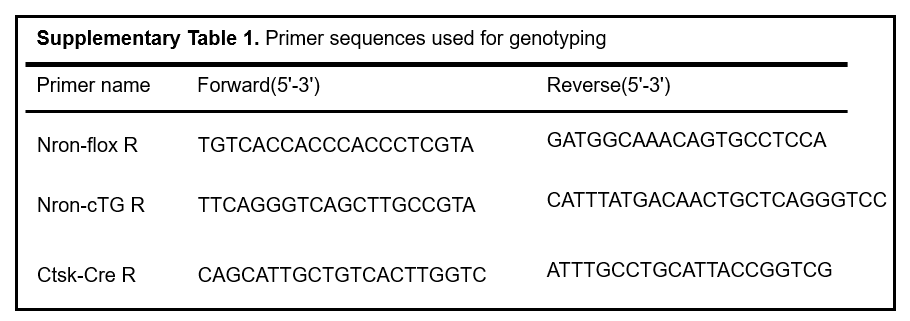


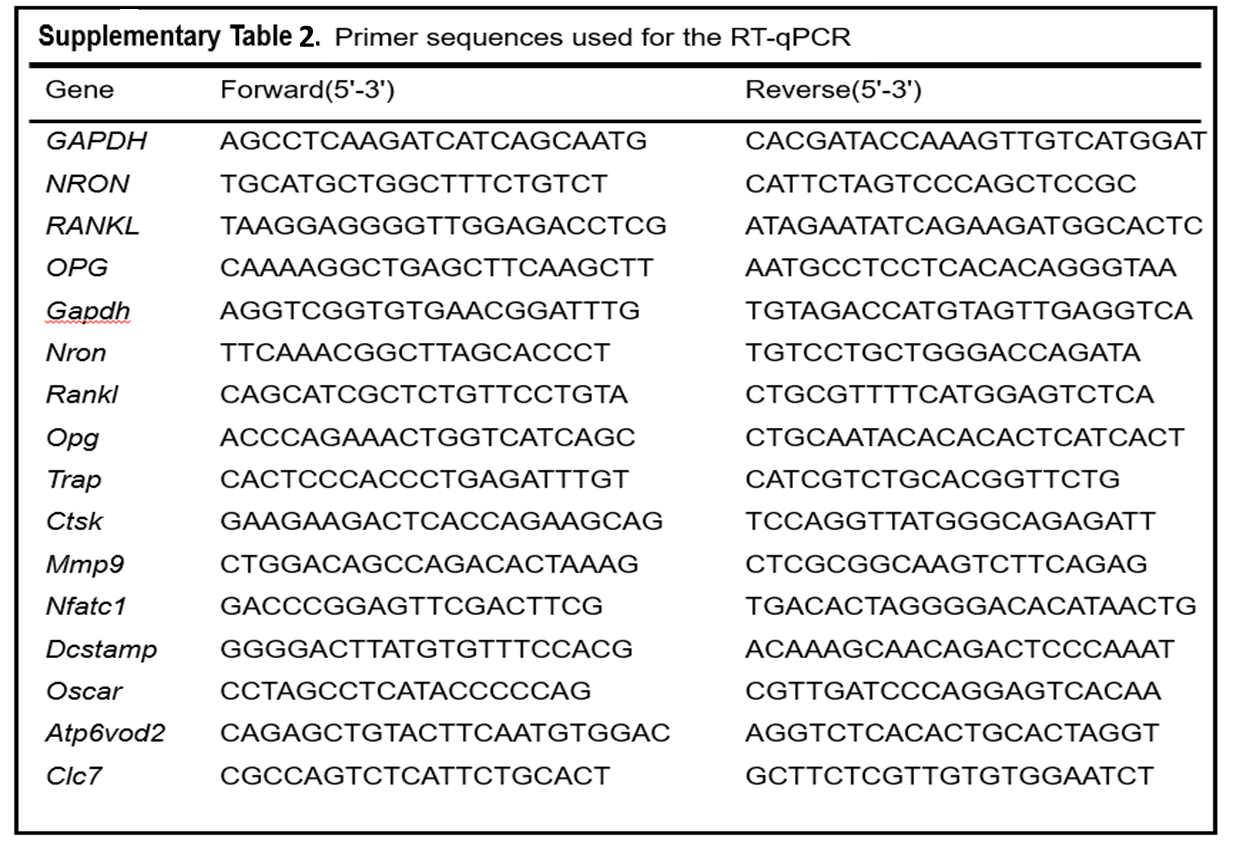

Supplement: Supplementary file 1 — Supplementary Information [file 41368_2020_77_MOESM1_ESM.docx]
